# Pneumococcal Tricuspid Valve Endocarditis in a Young African American: A Case for Inclusion of African Americans in Pneumococcal Vaccine Criteria

**DOI:** 10.1155/2010/982521

**Published:** 2010-09-05

**Authors:** Oghenerukevwe Odiete, Olagoke Akinwande, John J. Murray, Joseph Akamah

**Affiliations:** ^1^Department of Internal Medicine, Meharry Medical College, 1005 Dr. D.B. Todd Jr. Boulevard, Nashville, TN 37208, USA; ^2^Department of Internal Medicine, Aultman Hospital, 2600 6th st SW, Canton, OH 44710, USA; ^3^Department of Cardiology, Meharry Medical College, 1005 Dr. D.B. Todd Jr. Boulevard, Nashville, TN 37208, USA

## Abstract

Following the development of penicillin, complications from streptococcus pneumonia such as endocarditis have become rare. However, certain independent risk factors such as cigarette smoking and being of African-American (AA) decent have been associated with a higher incidence of invasive pneumococcal disease, but only cigarette smoking has been targeted by current recommendations from the Advisory Committee on Immunological Practices (ACIPs). We report a case of a young AA smoker, who developed an isolated tricuspid valve pneumococcal endocarditis. This case will illustrate the high susceptibility for invasive pneumococcus sequelae in AA, thereby raising the argument for the consideration of AA in the Pneumococcal Conjugate Vaccine (PCV) criteria, regardless of smoking history.

## 1. Introduction

Isolated tricuspid valve endocarditis is usually seen in intravenous drug users and patients with abnormal or prosthetic heart valves [1]. Since the introduction of penicillin, streptococcus pneumoniae has been an uncommon cause of bacterial endocarditis accounting for less than 5% of all the cases of endocarditis [2]. Cigarette smoking and being of an AA ethnicity are strong independent risk factors for invasive pneumococcal disease in the nonelderly and immunocompetent patients [3]. The rate of invasive pneumococcal disease in AA of all ages has at least twice the risk in the white population in the United States [4, 5]. Pneumococcal bacteremia could lead to complications such as endocarditis [6]. We report a case of an isolated tricuspid valve endocarditis due to PSSP in a nonelderly AA chronic smoker without any other additional risk factors.

## 2. Case Presentation

A 27-year-old African-American female, presented with complaints of cough and vomiting for the past two weeks. The patient was seen in the emergency room one week prior with similar problems and was sent home on oral levafloxacin and ibuprophen for CAP. Despite being on oral antibiotics, the patient did not improve and returned to the hospital with similar complaint in addition to a fever and a cough with whitish-yellow sputum production. The patient did not report any history of surgical, dental, or genitourinary procedures prior to the emergence of her symptoms. She also had no history of congenital cardiac disease, systemic lupus erythematosus, or rheumatic fever. She smoked one pack of cigarettes per day for 11 years, occasionally used marijuana and had no history of alcohol or intravenous drug use. 

On physical exam, her temperature was 102.8 F, heart rate was 98 bpm, respiratory rate was 34 rpm, and her blood pressure was 157/97 mmHg. On heart auscultation, the rhythm and rate were normal. S1 and S2 sounds were present. There was a grade 2/6 mid-systolic murmur heard best in the left lower sternal border, nonradiating, which was new when compared with the previous visit to the emergency department. The point of maximal impact (PMI) was nondisplaced. On lung exam, faint crackles were heard on the right side. The remaining physical examination was within the normal limit. 

**Figure 1:**
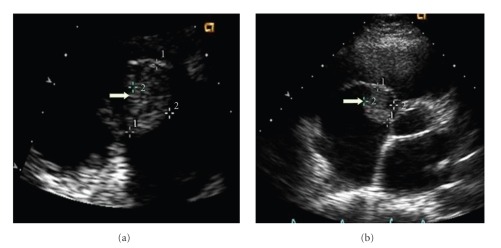
This is a transthoracic echocardiogram. White arrows point to the 2.6 × 1.7 cm pedunculated vegetation.

The laboratory results showed a white blood cell count of 26.1 k/cumm, hemoglobin 10.7 g/dL, and hematocrit 31.3%. The Urinalysis and urine drug screen was negative. Two separate sets of blood cultures were positive for Streptococcus pneumonia sensitive to penicillin (MIC <0.03). The Sputum culture was negative. The chest X-ray showed a right middle lobe infiltrate. Trans-thoracic echocardiogram (Figure 1) showed a 2.2 × 1.6 cm pedunculated vegetation, noted on the base of the posterior cusp of the tricuspid valve with moderate tricuspid regurgitation. The ejection fraction was >55%. The diagnosis of PSSP endocarditis was made and the patient was started on IV ceftriaxone. 

**Figure 2:**
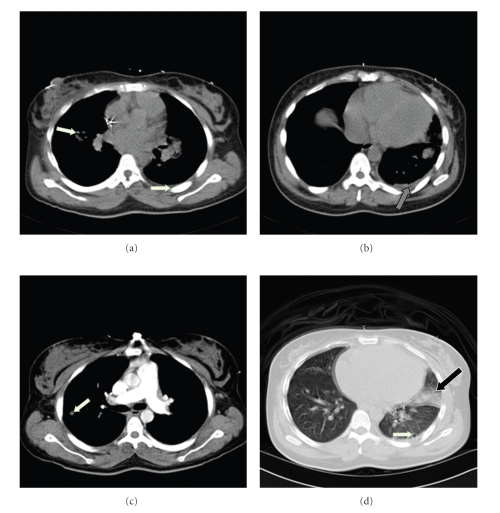
White shows cavitary and non-cavitary small nodular lesions changes consistent with pulmonary septic embolization and the black arrow shows a small left pleural effusion. Black and white arrow is showed a complicated ligulae pneumonia.

On the second day of admission, the patient started complaining of left pleuritic chest pain, dyspnea, and hemoptysis. Given the fact that the patient had valvular vegetations, a CT scan (Figure 2) was done to rule out pulmonary septic embolism. The CT scan showed cavitary and noncavitary small nodular lesions consistent with pulmonary septic embolization and a lingular pneumonia. After seven days of treatment, pyrexia persisted and gentamicin was added to the regimen. Patient remained afebrile for the next 72 hours and was discharged to complete a six-week course of antibiotics.

## 3. Discussion

The risk for invasive pneumococcal disease has been shown to vary between ethnic groups. Previous studies have identified African-American ethnicity as an independent risk factor for invasive pneumococcal disease with odds ratio of 3.4 in a study by Nuorti et al. [3] and of 4.6 in a study by Lexau et al. [4]. The rate of invasive pneumococcal disease in AA of all ages is about twice more frequent than in the white population in the United States [5, 6]. Although, cigarette smoking has been noted as a strong risk factor for invasive pneumococcal disease, the fact that this patient is AA significantly increases her overall risk.

A mechanism to explain the etiology of endocarditis in this young AA smoker could be that the patient had nasopharyngeal pneumococcal colonization, which is usually increased in both AA and smokers [7]. Following breach of the mucosal barriers—perhaps due to a viral infection—the patient developed invasive disease (bacteremia), and then secondary spread to the tricuspid valve. It is also conceivable that the invasive pneumococcus was either causative or as a result of the patients' pulmonary infection. Pneumococcal bacteremia can occur in patients with or without pneumococcal pneumonia and it can also occur in both immunocompetent and immunosuppressed individuals.

Most cases of isolated endocarditis of the tricuspid valve have been associated with intravenous drug use, intracardiac catheterization, cardiac anomalies, immunodeficiency, and indwelling central venous lines [1]. Tricuspid valve lesions tend to be attributed to Staphylococcus infections [8, 9] with pneumococcus being an uncommon causative organism [10]. In a review article of 197 patients with pneumococcal endocarditis, 10 patients (8.3%) had tricuspid valve involvement [11]. What made our patient unique are not the individual diagnostic findings but the aggregate clinical picture. This patient had isolated right heart involvement, which is less common than left-sided lesions. Also, the organism involved in this patient was penicillin sensitive pneumococcus (penicillin resistant species are more commonly reported [12, 13]). Furthermore, the patient also lacked the common risk factors frequently associated with tricuspid valve disease, which include chronic alcoholism and heart valve abnormalities. 

Due to the fact that pneumococcal bacteremia can lead to such serious complications such as endocarditis, which carries significant morbidity and mortality, it should be treated with vigilance. The Advisory Committee on Immunological Practices (ACIPs) recommends that Alaska Natives and American Indians of all ages should receive pneumococcal polysaccharide vaccine, if they are living in area in which the risk of invasive pneumococcal diseases is increased [14]. Current provisional recommendations do not include African-American adults aged <65 years without underlying illnesses. Even though we concede that this patient would have qualified for the vaccine because she is a smoker, we can also argue that evidence suggests she is a candidate by virtue of simply being AA. The cumulative risk of being an AA smoker should be treated with more vigilance and when these patients are identified in our clinics and emergency rooms. We must be assertive in encouraging them to receive vaccinations to prevent serious illness. PCV have resulted in the decrease of invasive pneumococcal disease in children, the elderly [15, 16], and should be considered for all patients at risk for invasive pneumococcal disease, which includes young AA.

## 4. Conclusion

Streptococcal pneumoniae is a rare cause of endocarditis, which rarely affects the tricuspid valve. A high index of suspicion for bacterial endocarditis should be applied in all patients, especially AA and smokers in the setting of pneumococcal bacteremia. Prudent auscultation for new murmurs in such patients must be executed judiciously. An echocardiogram might be indicated in a patient with pneumococcal pneumonia who is not responding to appropriate antibiotics to rule out endocarditis. Finally, it is worthy of debate that the inclusion of African Americans to the PCV vaccination criteria be considered to prevent the occurrence of pneumococcal bacteremia and its complications.

##  Competing Interests

The authors declare that they have no competing interests.

##  Authors' Contributions

O. Odiete conceived the study, substantially involved in the acquisition of data, compilation of relevant literature, and drafted the preliminary and final paper. O. Akinwande was involved intellectually in the revision, formatting and proofreading the paper. J. Murray was involve in some relevant literature compilation, and proofreading of the paper. J. Akamah was the cardiologist on consult, read the echocardiogram, and was involved in the provisional and final diagnosis. He also reviewed the paper. All authors have read and approved the final version of the paper.

##  Consent

Written informed consent was obtained from the patient for publication of this case report and accompanying images. A copy of the written consent is available for review by the Editor-in-Chief of this journal.
